# Priming for welfare: gut microbiota is associated with equitation conditions and behavior in horse athletes

**DOI:** 10.1038/s41598-020-65444-9

**Published:** 2020-05-20

**Authors:** Núria Mach, Alice Ruet, Allison Clark, David Bars-Cortina, Yuliaxis Ramayo-Caldas, Elisa Crisci, Samuel Pennarun, Sophie Dhorne-Pollet, Aline Foury, Marie-Pierre Moisan, Léa Lansade

**Affiliations:** 1 Animal Genetic and Integrative Biology, INRAE, University of Paris-Saclay, AgroParisTech, 78350 Jouy-en-Josas, France; 2 PRC, INRAE, CNRS, IFCE, University of Tours, 37380 Nouzilly, France; 3 Health Science Department, Open University of Catalonia, 08018 Barcelona, Spain; 4 Medicine Department, University of Lleida, 25001 Lleida, Spain; 5 Animal Breeding and Genetics Program, Institute for Research and Technology in Food and Agriculture (IRTA), Torre Marimon, 08140 Caldes de Montbui, Spain; 6 Department of Population Health and Pathobiology, College of Veterinary Medicine, North Carolina State University, Raleigh, NC 27607 USA; 7 US UMR 1426, INRAE, Genomic platform, 31326 Castanet-Tolosan, France; 8 University of Bordeaux, INRAE, NutriNeuro UMR 1286, 33076 Bordeaux, France

**Keywords:** Microbiology, Neuroscience, Systems biology, Behavioural methods

## Abstract

We simultaneously measured the fecal microbiota and multiple environmental and host-related variables in a cohort of 185 healthy horses reared in similar conditions during a period of eight months. The pattern of rare bacteria varied from host to host and was largely different between two time points. Among a suite of variables examined, equitation factors were highly associated with the gut microbiota variability, evoking a relationship between gut microbiota and high levels of physical and mental stressors. Behavioral indicators that pointed toward a compromised welfare state (*e.g*. stereotypies, hypervigilance and aggressiveness) were also associated with the gut microbiota, reinforcing the notion for the existence of the microbiota-gut-brain axis. These observations were consistent with the microbiability of behaviour traits (> 15%), illustrating the importance of gut microbial composition to animal behaviour. As more elite athletes suffer from stress, targeting the microbiota offers a new opportunity to investigate the bidirectional interactions within the brain gut microbiota axis.

## Introduction

The gut microbiota has become an increasingly popular area of study due to its role in host physical and mental health and metabolism^1,2^.

The horse gut microbiota consists of approximately 10^9^ microorganisms per gram of ingesta in the cecum^3^ including at least 108 bacterial genera^4–6^ and seven phyla^6–11^, as well as a myriad of protozoa, archaea and anaerobic fungi^12–14^. The horse gut microbiota promotes digestion and nutrient absorption for host energy production^15^, short chain fatty acid production^16,17^, and immune health such as protecting against pathogens and disease^14,18^.

The variation in the gut microbiota composition and functions in healthy equine athletes is not yet clear. For example, it is unknown whether inter-individual variation results from a continuum of different prevalent and rare bacterial compositions or whether individual gut microbiota gather around some stable communities that can be associated with health and well-being. Another hurdle is the lack of knowledge about the temporal stability of these holobionts, defined as individual hosts and their associated symbiotic microbial communities, and whether its stability might be associated with environmental and host-related variables that operate within gut microbiota. Studying such questions is complicated due to the complexity of varying genetic background as well as physiological and environmental conditions between and within horse athletes.

Strong evidence exists that diet is the major environmental factor contributing to gut microbial variation in horses^11,13,19–24^. Furthermore, the abundance of different species has been reported to vary dramatically based on host-specific factors like co-colonization with enteric parasites^14,25^, pathobionts^7,26–29^ and host genetics^30^. Yet, in addition to biotic stressors, equine gut microbiota dynamics have been associated to abiotic stressors such as exercise^13,31,32^. In fact, the gut microbiota composition has been associated with blood metabolites related to lipid metabolism and glycolysis at basal time as well as oxidative stress in endurance horses^13^, suggesting that physical exertion and stress may have a significant yet temporal effect on the gut microbiota composition and host metabolism. Moreover, endurance exercise has been shown to affect horse blood metabolome^33^, in addition to blood transcriptome and microtranscriptome^34,35^, suggesting that the type and intensity of physical training may also affect gut microbiota composition.

Just as physical stress can affect the gut microbiota, mental stress may also affect the equine gut-brain axis. Horses have been domesticated for thousands of years and have foraged in herds as animals of fight or flight. Welfare management (*e.g*. early and abrupt weaning, feed frequency, bedding, housing isolation versus foraging, time ridden, etc.) can have a significant effect on the horse’s overall well-being^36,37^ and contribute to inter-individual and temporal variations in equine gut microbial community structures^8,11,38^. Antwis and colleagues^39^ investigated how spatial and structural interactions influence the gut microbiome of semi-feral Welsh ponies compared to isolated individuals. They concluded that individual variation contributed to 52.6% of the gut microbiome followed by interactions and relationships among the group and social behavior, such as grooming. Modern stabling on the other hand, includes limited *ad libitum* feeding and turnout and isolation which can cause aberrant, repetitive behavior to develop that appear to have no purpose, known as stereotypies, including cribbing and weaving, as well as hypervigilance or more aggressive behaviors such as kicking and biting^40–42^. In this line, Destrez *et al*.^19^ evidenced a relationship between overall microbiota composition and occurrence of stress-related behaviors in horses. Precisely, the concentration of amylolytic bacteria and the *Succinivibrionaceae* relative abundance positively correlated to bowing (considered as an alert or alarm type of behavior) following a low fiber but high starch diet.

Although the aforementioned studies have shed light on the dynamics of the equine gut microbiome in health and stressful situations, the relative strengths of these various environmental and host-related variables, and their interactions, remain unclear, owing to the lack of systematic analysis that monitor both host and environmental variables in healthy sport horse populations that share a similar environment. Such analysis requires manageable systems wherein environmental and host-related variables and temporal variations in the gut microbiota can be monitored.

We have previously reported that breed exerted limited effect on fecal microbiota composition in a large cohort of healthy sport horses, from the same riding school, which we monitored for over an eight-month period^30^. However, the influence of multiple environmental and host-related variables (other than breed) were not assessed in this large-scale cohort study. In the present study, we sought to examine the influence of environmental (equitation, diet, housing, season) and host-related (*e.g*. age, sex, body condition, reproductive status, parasite infection status, pH, protozoa and fungi loads, behavioral indicators of a compromised welfare state and hematology) variables on fecal microbiota composition. Using these data, we first aimed to study inter-individual and temporal dynamics of fecal microbiota among athletes, and then we sought to study the association between the aforementioned variables related to environmental or host and the gut microbial community variation between and within-individuals over time. Lastly, we determined the covariation of microbial communities and host phenotypes through the microbiability (m^2^), that is, the cumulative effects the gut microbiome has on a particular phenotype.

## Results

### Cohort characteristics

**Figure 1:**
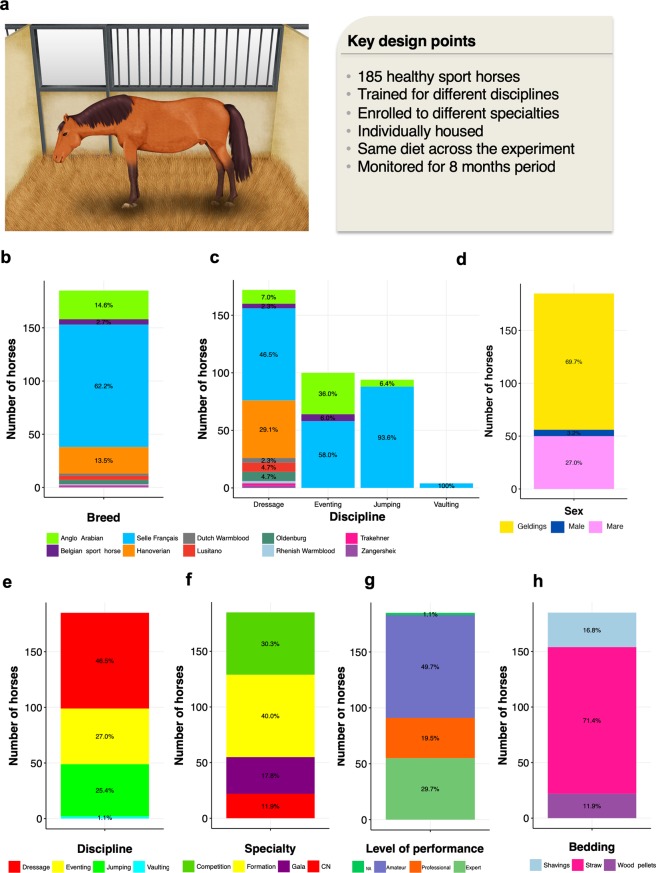
Description of the cohort. (**a**) Key points of the experimental design; (**b**) Bar plot representing the number of individuals ascribed to each breed; (**c**) Bar plot representing the number of individuals ascribed to each breed and discipline. For each discipline, the bar plot represents the number of individuals pertaining to each breed; (**d**) Bar plot representing the number of individuals ascribed to each sex; (**e**) Bar plot representing the number of individuals ascribed to each sport discipline; (**f**) Bar plot representing the number of individuals ascribed to each specialty: CN (Cadre Noir); (**g**) Bar plot representing the number of individuals qualified for each level of performance: NA (unknown), (**h**) Bar plot representing the number of individuals housed in straw bedding material, shavings or wood pellets. Written permission for publication of the drawing in the figure has been taken.

From the same cohort reported by Massacci *et al*.^30^, we selected 185 healthy sport horses cared for under similar conditions (Fig. 1a). A total of 180 individuals were shared in common between both studies. Fecal samples with time-matched blood, and behavior indicators were analyzed throughout a period of eight months apart. The cohort encompassed a large range of age, sex, athletic disciplines and performance (Supplementary Table S1).

The cohort in the present study was predominantly composed of « Selle Français » geldings with an average age of 10 ±  3.4 years (mean ± SD; Fig. 1b–d). Forty-six percent of our individuals were trained for dressage discipline, whereas another 25% were selected for show jumping and eventing disciplines, respectively (Fig. 1e). Overall, 30% of the individuals were enrolled to specialties with higher mental and physical engagements, that is, Gala and Cadre Noir (CN) specialties (Fig. 1f). Around 50% were engaged in a low level of performance, whereas 30% were regularly participating in high-level international competitions and 20% were trained to achieve the highest level of performance but did not participate in competitions (Fig. 1g). Concerning the body score condition, 78% of individuals were categorized as ideal (score from 3 to 3.25), whilst 14% presented overweight (score from 3.25 to 5) and 8% were thin (score < 3). The average horse feed units (UFC) was 2.9 ± 0.64 (per kg dry matter), whereas the estimated daily intake of protein and fiber from concentrate was around 400 ± 80 (g/ per kg dry matter; Supplementary Table S1). While all horses were restrictively housed in individual stalls, 71% were kept in straw bedding instead of shavings or wood pellets (Fig. 1h).

**Figure 2:**
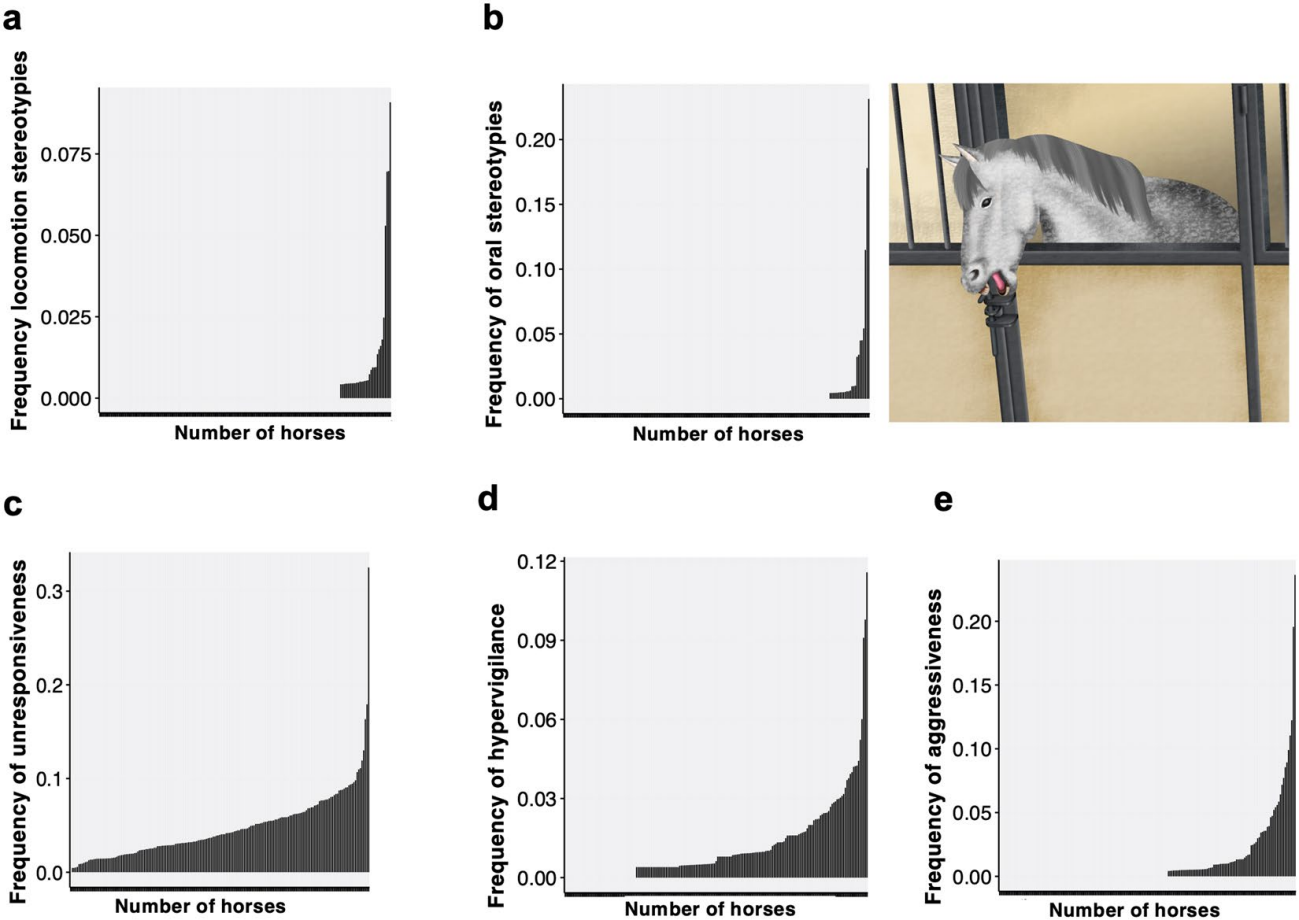
Frequency of behaviors related to welfare impairment in each of the 185 individuals during the eight-months period of observations. (**a**) Histogram showing the frequency of scans of locomotion stereotypies; (**b**) Histogram showing the frequency of scans of oral stereotypies; (**c**) Histogram showing the frequency of scans of unresponsiveness to the environment; (**d**) Histogram showing the frequency of scans of hypervigilance; and (**e**) Histogram showing the frequency of scans of aggressiveness behavior. Written permission for publication of the drawing in the figure has been taken.

The values of cellular components of the blood fell within the normal reference range values of clinically healthy horses (Supplementary Table S1). Behaviour assessment showed that the expression of both oral and locomotion-related stereotypies was observed in 17% of the individuals (Fig. 2a,b), whilst 99% of the individuals were experiencing unresponsiveness to the environment (the so-called withdrawn posture) at least once within the eight-month period (Fig. 2c). Hypervigilance was observed in 75% of our individuals (Fig. 2d), whereas aggressiveness was observed in 43% of our horses (Fig. 2e). Further information on equitation, environmental and host variables is depicted in Supplementary Table S1.

### The fecal microbiota of athletes reared in similar conditions

The fecal microbial communities of the 185 horses were tracked at T1 and T2 (eight months later). Each fecal sample was characterized via next-generation sequencing of V3–V4 hyper variable region of the 16 S rRNA gene, allowing for a total of 13,012,999 high-quality sequences reads (mean per subject: 35,170 $\pm \,$11,875; range: 10,374–109,131; Fig. S1). Reads were clustered into 15,154 chimera- and singleton-filtered operational taxonomic units (OTUs) at 97% sequence similarity (Supplementary Tables S2 and S3).

**Figure 3:**
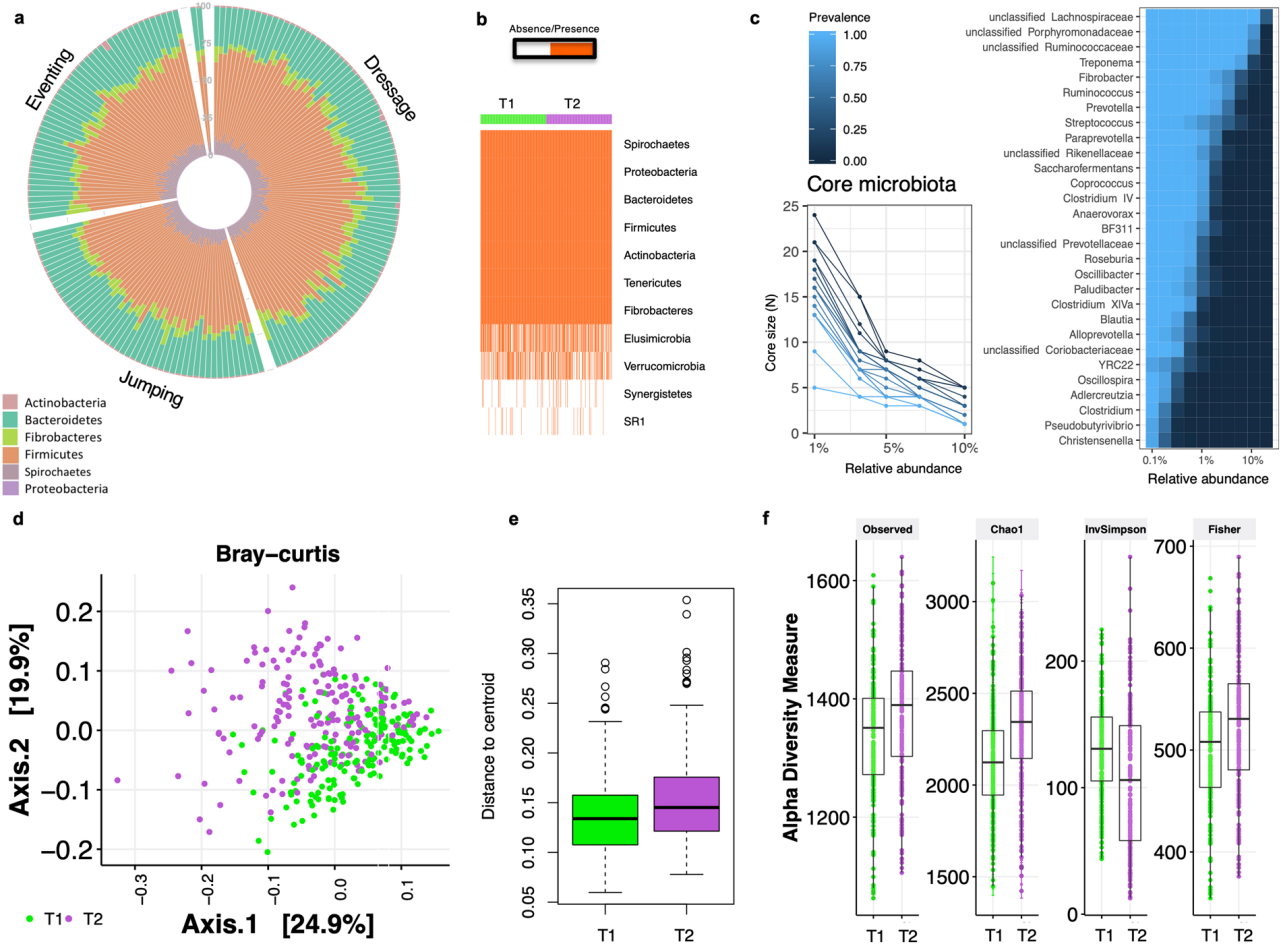
Inter-individual and intra-individual gut microbiota variations. (**a**) Circular stacked bar plot of the main phyla for each individual in the cohort. Rare phyla such as Elusimicrobia, Verrucomicrobia and Synergistetes are not represented (mean relative abundance across T1 and T2 < 0.01%). Individuals are grouped by discipline; (**b**) Matrix showing the presence or absence of the phyla detected in our cohort. Each entry in the matrix indicates the presence or absence of each phylum in each individual. Individuals are grouped by time point; (**c**) The difference in number of genera and their prevalence at different relative abundance thresholds, and the heatmap depicting the core microbiota and their prevalence at different detection thresholds. The heatmap displays the genera shared by 99% of individuals in the cohort with a minimum detection threshold of 0.001%; (**d**) Dissimilarities in fecal microbiota composition represented by the non‐metric multidimensional scaling (NMDS) ordination plot, with Bray–Curtis dissimilarity index calculated on un-scaled OTU abundances. Samples are colored by time point: T1 (green), and T2 (purple); (**e**) Variation among the two time points as indicated by dispersion, that is, the distance of individuals from the centroid of their time point. Boxes show median and interquartile range, and whiskers indicate 5^th^ to 95^th^ percentile; (**f**) Box plot representation of the α‐diversity indexes using the rarefied OTU table for each time point. The box plots feature the median (center line) and interquartile range, and whiskers indicate 5^th^ to 95^th^ percentile.

The inter-individual and intra-individual gut microbiota variations were reconstructed across subjects and time, based on the variation of OTU, phyla and genera abundances. The non-multiple dimensional scaling (NMDS) based on weighted UniFrac distance or Bray-Curtis dissimilarity did not show a community structure or enterotype-like clusters in our cohort. Individuals did not fall into distinct bins based on their taxonomic similarity. However, there were apparent dynamics of the microbial signatures that characterized each individual. At the OTU level, the average number of within-individual differences was around eight-fold smaller than the average number of differences between unrelated hosts (Analysis of similarity (ANOSIM), R = 0.16, *p* = 9.99 e^−05^, range of fold change from 1.01 to 1,031). Even at phylum level, there was a seeming individual effect on the microbial signatures (Fig. 3a). On average, the between-individuals divergence was 2.29 times greater than the within-individual divergence (ANOSIM, R = 0.30, *p* = 0.001). Specifically, rare phyla such Elusimicrobia, Verrucomicrobia and Synergistetes showed high rates of divergence between hosts and the two time points (Fig. 3b), whereas the Gram-negative Bacteroidetes and Proteobacteria, and Gram-positive Firmicutes remained more stable. This trend was bolstered at the genus level. While several prevalent genera (*e.g. Coprococcus, Clostridium XIVa, Treponema, Saccharofermentans* and *Anaerovorax*) presented low divergence rates between hosts (Supplementary Table S4), we note that this pattern was not universal. Most of the rare genera showed higher inter-individual variability, including multiple α-, ε- and γ-proteobacterial genera such as *Sphingomonas, Ruminobacter* and the pathobionts *Escherichia, Helicobacter, Bilophila* and *Pseudomonas* (Supplementary Table S4). The observed individual microbiota profiles at phylum and genera level are depicted in the Supplementary Fig. S2.

Notwithstanding the observed individuality, combined, the intestinal microbial community of the 185 individuals yielded a microbiota core of 29 genera (*e.g*., the genera shared by 99% of all sampling events with a minimum 0.1% mean relative abundance). Overall, 52% of the core genera belonged to the Firmicutes phylum, and mainly to the *Lachnospiraceae, Porphyromonadaceae* and *Ruminococcaceae* families (Fig. 3c). The majority of these genera (90%) were among the 30 highest abundant (Supplementary Fig. S2) accounting for more than 75% of the sequences in the data.

### Temporal dynamics of the fecal microbiota

Although the within-subject distances were lower than between subject distances, the microbial temporal community dynamics were especially apparent (multivariate analysis of variance with permutation (PERMANOVA) based on the Bray-Curtis dissimilarity, R = 0.038, adjusted *p*-value = 9.99 e^−05^; Fig. 3d). The median Bray-Curtis dissimilarity after eight months (T2) was about 2.25% greater than the initial value (T1, ANOVA; adjusted *p-*value = 1.93 e^−05^; Fig. 3e). The higher heterogeneity among individuals at T2 was particularly associated with overgrowth of rare taxa (increased number of observed species) and increased Chao1 and Fisher diversity indexes (adjusted *p* < 0.05, Wilcoxon test, Fig. 3f).

**Figure 4:**
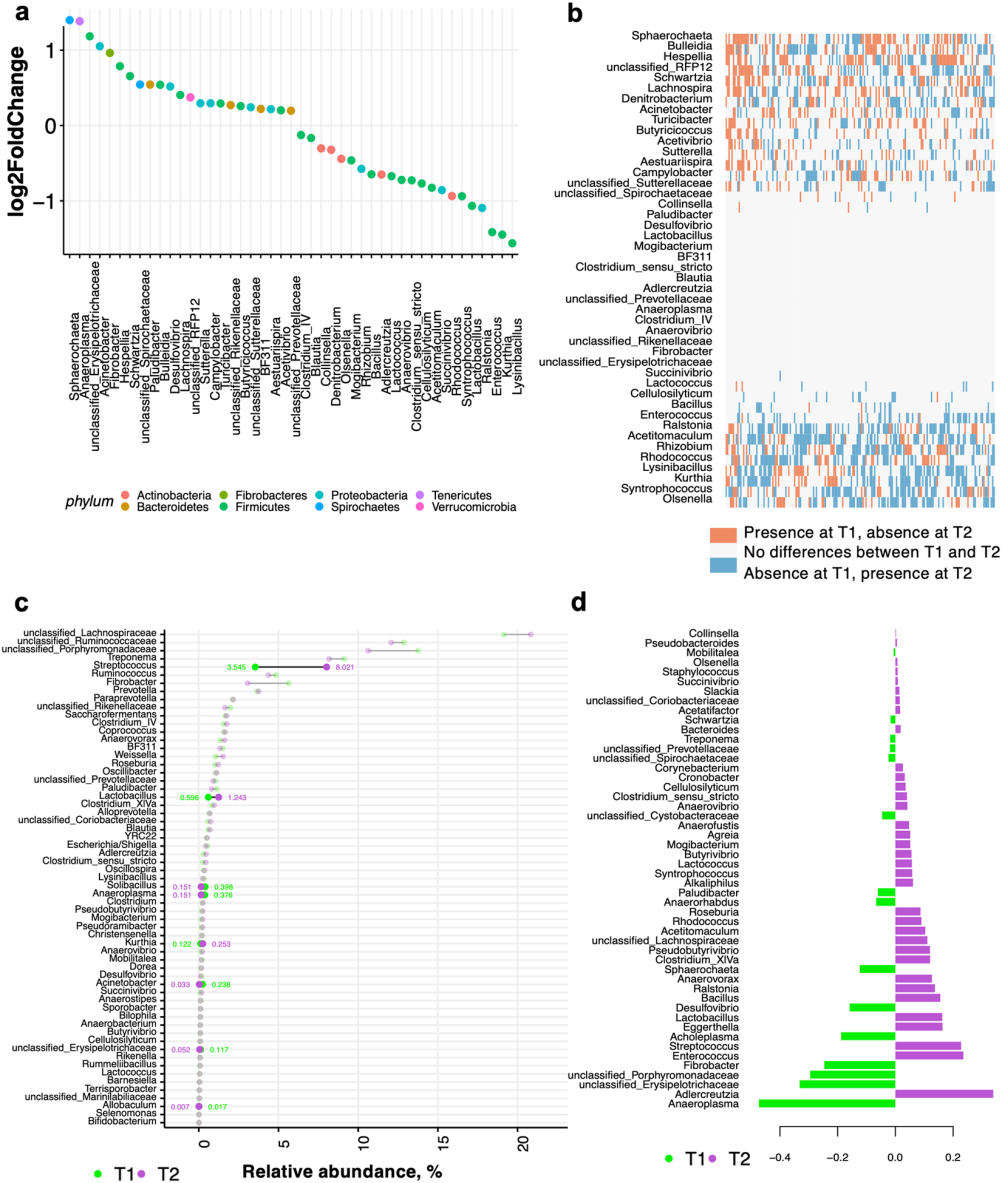
Dynamics of gut bacterial genera between the two time points. (**a**) Dot plot representation of log-transformed fold change of genera that were significantly different between T 1 and T2, as reported by DESeq2 model. The logs of fold changes lying between 0 and 2 indicate that genera were more abundant in T1 than T 2. By contrast, the logs of fold changes lying between 0 and −2 indicate that the genera abundances were lower in T1 compared to T2. Dots are colored by phyla; (**b**) Matrix showing the presence or absence of the differentially expressed genera detected by DESeq2 model. Each entry in the matrix indicates the presence or absence of each genus in each individual and time point. In the heatmap, red = presence at T1 and absence at T2, white = no differences between T1 and T2, and blue = absence at T1 and presence at T2; (**c**) Taxa with the largest variation in abundances between the two time points, as reported by the similarity percentage procedure (SIMPER). Each point represents the genera relative abundance at T1 or T2. The bars show the variance interval around the two time points. Points are colored by time points: T1 (green) and T2 (purple). Unstable genera across time (>50% of variation) are highlighted in bold; (**d**) The partial least squares discriminant analysis (PLS-DA) loading plot shows the contributing genera at each time point.

The microbiota temporal dynamics were further analyzed by evaluating the genera propensity to variation over two time points, as the magnitude of change above or below the average relative abundance. The relative abundance of 45 genera was significantly different between the two time points (adjusted *p-*values < 0.05 with DESeq2; Table S5, Fig. 4a). The taxa most differentially abundant at T1 compared to T2 primarily belonged to the family *Erysipelotrichaceae* (log_2_ fold change = 1.18, adjusted *p*-value = 2.88 e^−203^), to the genera *Anaeroplasma* (log_2_ fold change = 1.30, adjusted *p* = 1.49 e^−84^) and to the genera *Enterococcus* (log_2_ fold change = -1.41, adjusted *p* = 2.43 e^−80^; Fig. 4a). Most of the abundant bacteria (prevalence > 1%) did not have any detectable differences between both time points, roughly eight-months apart. Yet, dominant bacteria such as *Fibrobacter, Clostridium* IV, BF311 and members of the *Rikenellaceae* family were highly different between T1 and T2 (Supplementary Table S5). In contrast, a larger fraction of rare genera (<1% prevalence) presented important differences in abundance between T1 and T2 (Fig. 4b). In line with these results, the similarity percentage procedure (SIMPER) also showed that some of the taxa with the largest variation in abundances between T1 and T2 were *Streptococcus* and *Lactobacillus*, as well as rare taxa such as *Solibacillus, Anaeroplasma, Kurthia, Acinetobacter* and the members of the yet not classified *Erysipelotrichaceae* (Fig. 4c). Based on the partial least squares discriminant analysis (PLS-DA) of genus-level community composition, the vast majority of discriminatory taxa between T1 and T2 belonged to the members of the yet not classified *Erysipelotrichaceae* and *Porphyromonadaceae* families, as well as *Anaeroplasma* and *Fibrobacter*, while the taxa discriminatory at T2 were *Adlercreutzia, Enterococcus, Streptococcus, Lactobacillus* and *Bacillus* genera (Fig. 4d).

Compositional changes between T1 and T2 were followed by functional implications, that is, a reduction of fecal pH and anaerobic fungal loads at T2 (Wilcoxon test, adjusted *p* < 0.05; Supplementary Fig. S3), with more than 0.5 log of difference between both time points. Conversely, bacteria and ciliate protozoa loads were significantly greater at T2 (Wilcoxon test, adjusted *p* < 0.05; Supplementary Fig. S3).

The observed microbial community dynamics coincided with a seasonal shift (Supplementary Fig. S4). A heat wave took place at T2, resulting in almost no rainfall (0.2 mm) and high temperatures peaking at 36.7 °C. However, no dehydration occurred in horses as supported by no significant differences in the hematocrit values between T1 and T2. No changes in food availability or quality were observed between time points.

### Association between host and environmental variables and the intra-individual and temporal dynamics of the fecal microbiota

**Figure 5:**
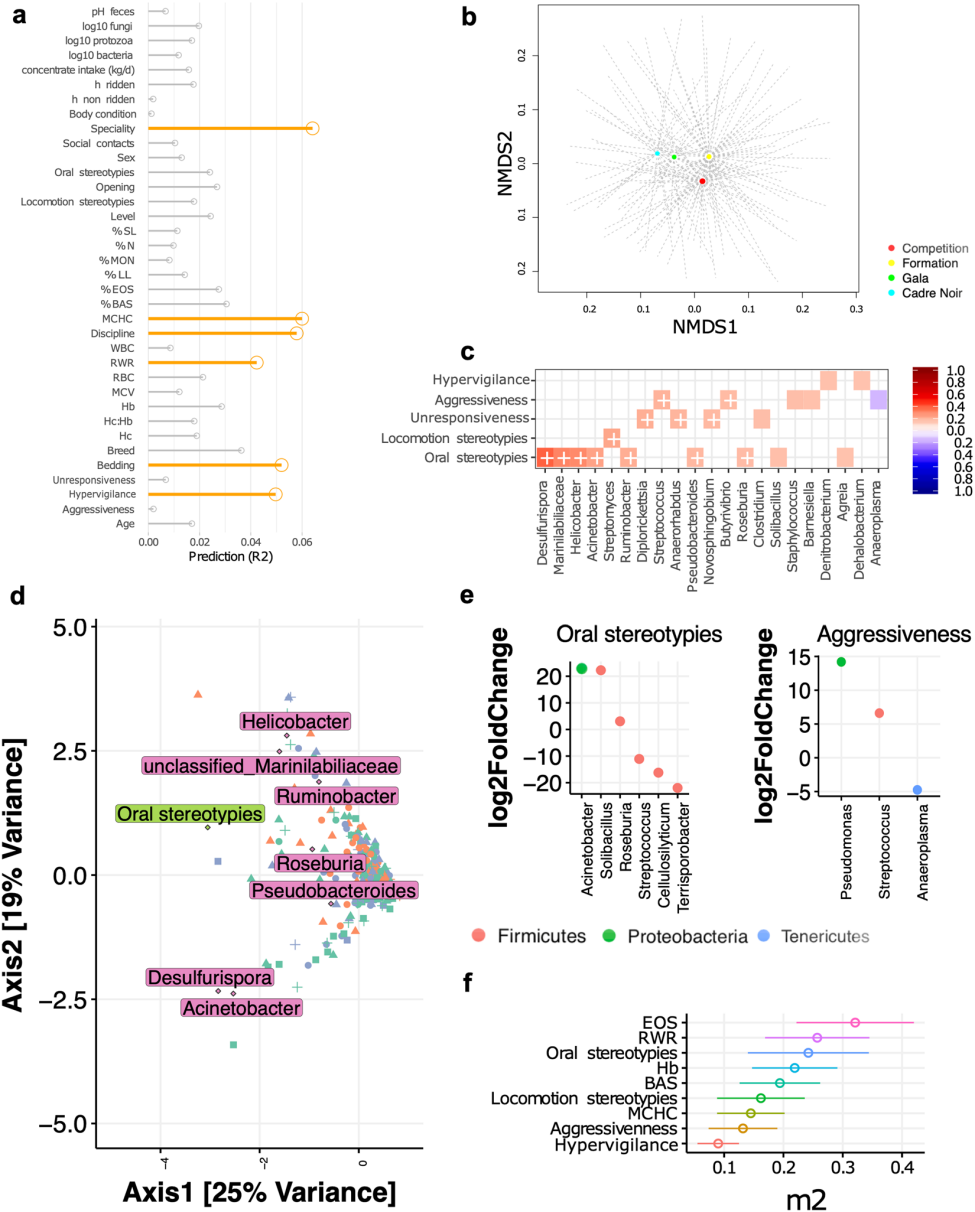
Associations between gut microbiota, host and environmental variables. (**a**) Effect size of covariates of fecal microbiota composition. Each bar shows the fraction of microbial variation explained in the model; (**b**) Dissimilarities in fecal microbiota composition represented by the non-metric multidimensional scaling (NMDS) ordination plot, with Bray–Curtis dissimilarity index calculated on un-scaled OTU abundances. Samples centroids are colored by specialty; (**c**) Cross-correlation matrix between behavior indicators and genera. Each entry in the heatmap is the correlation coefficient of a particular genus and a behavior indicator. In the heatmap, red = positive correlation values, blue = negative correlation values. Color intensity is proportional to the correlation coefficients. In the right side of the correlogram, the legend color shows the correlation coefficients and the corresponding colors. Correlations with adjusted *p* < 0.05 are considered significant. In these cases, white crosses are added in the corresponding cell. The correlations with *p* > 0.10 are leaved blank in the matrix; (**d**) Canonical Correspondence Analysis (CCA) ordination plot of genus table, and results of the analysis of host variables affecting bacterial divergence. Significant host variables are indicted as rectangles, and genera associated to the correspondent significant variable are depicted in diamonds with text labels. Points, triangles, squares and crosses represent the specialty, whereas the color of the features represent the discipline; (**e**) Dot plot representations of log-transformed fold change of genera that were significantly different between horses expressing oral stereotypies or aggressiveness. In both graphs, the logs of fold changes superior to 0 indicate that genera were more abundant in individuals expressing the behavior indicator than individuals not showing the behavior at any time of the experiment. By contrast, the logs of fold changes inferior to 0 indicate that the genera abundances were lower in individuals expressing the behavior indicator. Dots are colored by phyla; (**f**) Microbiability estimates. Each microbiability estimate is expressed as a percentage of the phenotypic variance and is followed by its standard error.

We then sought to determine whether the observed microbiota divergence was associated with environmental variables, host-related variables or a combination of both. We simultaneously measured 41 metadata variables, including multiple environmental (diet, bedding, housing, equitation factors) and host-related variables (*e.g*. age, sex, parasite infection status, behavior, hematology, fecal pH, loads of fungi and protozoa; Supplementary Table S1). Individuals were sampled at two time points to tease apart the relative influence of environmental and host variables. Using these data, we examined the association between extrinsic and intrinsic variables and the gut microbial community divergence. The feature-selection algorithm selected six main variables that together inferred over 32% of the variance in microbiota β-diversity (adjusted *p* < 0.05; Fig. 5a). The aforementioned variables were the equitation specialty and discipline, the type of bedding, hypervigilance behavior, and hematological measures such as mean corpuscular hemoglobin concentration (MCHC) and erythrocytes to leukocytes ratio (RWR).

The calculation of the covariates’ effect size revealed that specialty had the largest explanatory power on microbiota composition (Fig. 5b), including 6.5% of community variation. Horses trained for Gala and CN events grouped together, showing that they shared a more similar fecal microbiota, whereas horses trained for competition or used for formation purposes were clustered apart (Fig. 5b). Specialty effect size was followed by equitation discipline (5.79% of the variation) and type of bedding (5.2% of variation). The NMDS, based on Bray-Curtis dissimilarity, clearly stratified communities of bacteria from individuals trained for dressage and jumping from other type of disciplines (Supplementary Fig. S5). Concerning the bedding material, what was remarkable was that the horses housed in stalls on straw bedding were clustered apart from those housed in stalls on shavings and wood pellets (Supplementary Fig. S5).

Beyond the variables outlined above, the composition of fecal microbiota was also effected by host variables such as MCHC (6% of the variation) and hypervigilant behavior (4.97% of variation). Oral stereotypies tended to influence (*p*-value = 0.06) the fecal microbiota composition, albeit accounting for lower values of variation (~ 2.5%).

Fecal pH, and loads of ciliate protozoa and anaerobic fungi did not trigger the overall microbiota divergence between individuals. Other events that are generally thought to affect microbiota composition were not associated with fecal microbiota differences in our study, including the daily quantity of high-starch grain cereals received. Similarly, this analysis failed to detect significant association for host variables such as age or sex.

In order to resolve any clear relationships between the hematological and behavior host-related variables and the gut microbiota, we performed an interaction analysis between the aforementioned covariates and the bacteria genera using Pearson correlation analysis adjusted for multiple comparisons. These data showed that the relative abundances of single gut bacteria were significantly associated with the expression of four behavioral indicators of a compromised welfare state, including oral and locomotion stereotypies, aggressiveness and unresponsiveness to the environment (Fig. 5c), but not with the blood erythron and leukon. Precisely, the abundance of *Desulfurispora, Helicobacter, Acinetobacter, Ruminobacter, Pseudobacteroides, Roseburia* and members of the yet not classified *Marinilabiliaceae* family was greater with higher occurrences of oral stereotypies, whilst *Streptomyces* was greater with higher incidence of locomotion stereotypies (adjusted *p*-value <0.05). Higher frequency of aggressiveness was positively associated with overgrowth of two other bacterial genera: *Streptococcus* and *Butyrivibrio* spp. (adjusted *p* < 0.05), but inversely correlated to the prevalence of *Anaeroplasma* (adjusted *p* < 0.10). The manifestation of unresponsiveness to the environment was positively linked to the prevalence of *Diplorickettsia, Anaerorhabdus* and *Novosphyngobium* (adjusted *p* < 0.05). Hypervigilance could not be significantly related to any specific taxa at an adjusted *p* < 0.05, but tended to be significantly associated with *Denitrobacterium* and *Dehalobacterium* (adjusted *p* < 0.10). Complementary, the sparse canonical correspondence analysis (CCA) confirmed that oral stereotypies strongly correlated to the prevalence of *Acinetobacter, Desulfurispora, Pseudobacteroides, Roseburia, Ruminobacter, Helicobacter* and members of the yet not classified *Marinilabiliaceae* family (Fig. 5d). The robustness of these identified links was further studied through the DESeq2 differential abundance comparison method, which led to similar observations. For example, horses expressing oral stereotypies harbored higher abundance of *Acinetobacter, Solibacillus* and *Roseburia* but lower abundance of *Streptococcus, Cellulosilyticum* and *Terrisporobacter*, whilst horses showing aggressive behaviors harbored significantly higher abundances of *Pseudomonas* and *Streptococcus*, but lower abundance of *Anaeroplasma* (DESeq2, adjusted *p* < 0.05; Fig. 5e).

### Microbiability of the host behavior and hematological variables

We further estimated the microbiability (m^2^) of the host variables traits reported as significantly associated with the microbiota variation in the previous section. The m^2^, by analogy with genetic heritability, quantifies the overall proportion of host phenotypic variance explained by the microbial community after accounting for breed, discipline and host genetics^43^. Larger m^2^ values indicate a higher microbial contribution with respect to the phenotype of interest. We obtained estimated m^2^ levels of 14.5% for MCHC, 19.4% for basophils, 21.9% for hemoglobin, 25.7% for RWR and 32.1% for the eosinophils (Fig. 5f). The microbiome was also strongly associated with behaviour indicators of a compromised welfare state. Interestingly, the m^2^ of oral and locomotion stereotypies, estimated individually, were 24.2% and 16.2%, respectively, whereas the estimated m^2^ of aggressiveness was 13% and that of hypervigilance was 9%.

## Discussion

Our previous work on the association between breeds and fecal microbiota in the same cohort of individuals demonstrated that breed exerted limited effects on fecal microbiota composition^30^. Until this current study, our prior work had been limited to describe the effect of one single variable (breed) on gut microbiota composition (Massacci *et al*.^30^), therefore neglecting the global interactions that occur across multiple environmental and host variables across time. Here, instead, we decided to explore the temporal dynamics of the fecal microbiota by simultaneously combining environmental variables together with host factors. Additionally, this study also led to new insight into the covariation of microbial communities and host phenotypes through the microbiability, that is, the cumulative effects the gut microbiome has on a particular trait.

First of all, this study showed that significant bacterial community drifts of the gut microbiota occurred when assessed at two time points approximately eight-month apart, and that the magnitude and significance of these drifts varied among horses. Similarly, Salem *et al*.^11^ contended that fecal microbiota in healthy horses was not stable over a 12-month time period. Therefore, the stability of fecal microbiota communities should not be assumed even in healthy individuals.

Our study also highlighted the intra-individual temporal instability of rare bacteria between the two time points, not allowing a horse to be identified by their unique microbial fingerprint up to 8 months later. For instance, we detected widespread colonization of rare taxa that contained multiple α-, ε- and γ-proteobacterial genera: including the potentially harmful pathobionts *Escherichia/Shigella*, *Helicobacter, Bilophila* and *Pseudomonas*. However, given that 16 S rRNA gene sequencing has a limited capacity to resolve the taxa to the species level and that there are limited studies about horse pathobiome, referred to as the set of associated microorganisms linked with reduced health status^44,45﻿^, we cannot exclude the fact these are commensal strains that likely constitute a large reservoir for diversity and functionality. One of the best approaches to improve the resolution of taxonomical classification would be the use of whole metagenome shotgun sequencing, which assure non-amplification bias and unbiased taxonomic annotation^46^. Moreover, because of the limited sequencing coverage (~30.000 reads per sample in average), the diversity of the rare microbial communities was likely underrepresented, as rarer taxa are less prone to be detected^47^. Greater number of individuals (*N*) and sequencing efforts are required to determine the rare taxa, which are increasingly recognized as drivers of key functions in host-associated microbiomes^48^. In spite of this drift, 29 genera (mostly known commensals) remained sufficiently stable, which allowed us to identify a core microbiota. The observed core microbiota was nearly identical to the one described by Massacci *et al*.^30^. Since a total of 180 individuals (out of the 185 used here) were shared by both studies, similarities in the core microbiota between both studies were expected. This persistent core microbiota accounted for a very large proportion of the sequences, consistent with previous horse studies^5,13,49^.

There were many factors that could have been potentially associated with the gut microbiota divergences observed between the two time points, including environmental (mainly diet), stochastic, genetic and epigenetic diversity, and a complex array of intra-host variables and co-morbidities^11,30^. While investigating the environmental and host-microbiota relationship on our considerably large data set, we demonstrated that gut microbiota divergence in healthy sport horses kept in the same riding school and fed the same diet was primarily related to equitation factors such as specialty and discipline, which had no significant evidence for temporal structuring. Of all the specialties in our cohort, Gala and Cadre Noir linked to gut microbiota composition the most probably due to the fact that the degree of the physical and mental stress during training and show events, as well as the stress of being transported to more events, was higher compared to other specialties. In agreement, as physical and mental stressors were likely the main variables associated with microbial community composition of our cohort, gut communities of individuals bred for dressage and jumping were more similar from one another than communities from individuals bred for eventing. In our study, the prevalence of stress was believed to be higher in horses that were trained for dressage and jumping, as most of them were elite athletes that travelled in international competitions, trained many hours per day, 5 days a week, for several weeks without taking time off from intense training. Accordingly to the review by Art and Lekeux^50^, elite sports horses frequently suffer from several problems related to either endogenous or exogenous stressors during their athletic career. The authors stated that these types of stressors are actually caused by the use of horses as competition “tools” combined with inadequate recovery and environmental variables such as living in closed boxes, eating dry concentrates and long-distance road transports. Therefore, we assumed that in our cohort the more horses were ridden, transported to events, competing in front of spectators and suffered from inadequate recovery, the more likely they were to experience physical and emotional stress. Recording hormonal indicators and symptoms associated with fatigue, performance decline, change in daily intake, weight loss, as well as inflammation and immunosuppression could have surely helped to obtain a better understanding of stress levels in our athletes. The aforementioned hypotheses are in line with those reported in military personnel, who commonly endure prolonged periods of physical exertion, psychological stress, sleep deprivation and environmental extremes during training and combat^51^. The authors suggested that a multiple-stressor environment characterized by high physical exertion, suboptimal energy intake, muscle damage, and inflammation adversely affects intestinal barrier integrity and alters intestinal microbiota composition and metabolism^51^. A key study in mice exposed to forced treadmill running for six weeks proved that the gut microbiota interacts directly with stress hormones in the mucosal layer of the gastrointestinal tract^52^.

Complementary to these findings, we observed that mean corpuscular hemoglobin concentration and erythrocyte to leukocyte ratio (both associated with blood oxygen uptake capacity) were associated with microbial community structures, with stronger effect size than other host variables such as age, sex and breed. During intense exercise, athletes’ body temperature increases and blood pools away from the gastrointestinal tract to periphery muscles and organs such as the heart and lungs during intense physical activity (reviewed by Clark and Mach^53^). The redistribution of blood flow away from the intestines together with thermal damage to the intestinal mucosa can cause the leakage of epithelium, followed by an inflammatory responses (reviewed by Clark and Mach^53^). As observed in the inflammatory bowel diseases, the leakage of the epithelium can cause entry of blood into the gastrointestinal tract, provoking the release of hemoglobin carrying oxygen in the intestinal mucosa and lumen where the gut bacteria reside^54^. Such increase in the oxygen gradient within the gastrointestinal tract are therefore likely to represent genuine selective advantage to the resident facultative anaerobes or potentially aerobes. Simultaneous changes in the oxygen gradient within gastrointestinal milieu in mice and humans have been reported to impact the gut microbiota^55^. In this manner, we hypothesized that high physical exertion and performance were accompanied by hyperthermia, ischemia and hypoperfusion in the intestinal tract, which could have caused intestinal barrier disruption, followed by a blood entry in the lumen and an alteration of the oxygen gradient.

Just as equitation factors were associated with the gut microbiota, behaviors related to mental distress, including hypervigilance and to a lesser extent, the oral stereotypies, were related to the composition of the fecal microbiota in our cohort. It is now clear that imbalances in the microbial composition of the gut are present in mental illnesses such as anxiety disorders^56,57^. Moreover, fecal microbiota transplantation from patients with depression to microbiota depleted rats transmitted the anxiety-like behavior^58^, suggesting that the gut microbiota may play a causal role in the development of depression and anxiety via the gut-brain axis. On the other hand, *Lactobacillus* spp. improved social exchanges in stressed mice^59^, and *Bacteroides* spp. ameliorated repetitive and anxiety-like behaviors and interaction impairments in mice, likely through restoration of a specific bacterial metabolite^60^.Therefore, gut microbiota could be a target in the treatment and prevention of hypervigilant behavior estimated in 75% of horses of all ages in our study.

Oral stereotypies, which indicate mental distress, could be a coping mechanism for horses in response to stress in their environment (*e.g*. movement restriction, limited forage intake and feeding frustration)^36^. Crib biting in horses has been associated with higher ghrelin levels^61^, a hormone produced by enteroendocrine cells in the gastrointestinal tract that not only regulates hunger and gut motility, but also anxiety, stress and fear like behaviors via gut-brain axis signaling (reviewed by Lach *et al*.^62^). Therefore, oral stereotypies might have presumably altered feeding patterns and the central nervous system responses, and consequently modified the gastrointestinal milieu (*e.g*. inflammation, acidosis, reduction of SCFA, redox potential, mucus thickness or bile salt abundance), and the gut microbiota composition. As reviewed by Clark and Mach^53^, the physical and emotional stress in elite athletes stimulates the sympatho-adrenomedullary (SAM) and hypothalamus-pituitary-adrenal (HPA) axes, resulting in the release of catecholamines (norepinephrine (NE) and epinephrine) and glucocorticoids into circulatory system. Stress also activates the autonomic nervous system (ANS), which provides the most immediate response to stressor stimulus through its sympathetic and parasympathetic arms, and increases the neuronal release of NE and other neurotransmitters in the gastrointestinal tract. NE has shown to directly promote the pathobiome, including *Aeromonas hydrophila, Bordetella* spp., *Campylobacter jejuni, Helicobacter pylori, Listeria* spp. and *Salmonella enterica spp*., among others^63,64^. For instance, some of the ways by which NE promotes pathobionts overgrowth is by facilitating *E. coli* adherence to the intestinal wall or activating the expression of virulence-associated factors in *Salmonella typhimurium*, which then makes infection by these bacteria easier^64^). In addition, the oral stereotypies indicator was moderately predictable from gut microbiota composition (microbiability ~ 25%), suggesting that the intestinal microbiota could be considered as an important metabolic organ with major relevance to horse behavior. Further support for this hypothesis has been elegantly reviewed by Johnson et colleagues^65^, who reported evidences that symbionts have evolved to manipulate host behaviour for their own ends. As genetics have been insufficient to explain the physiology of horses with extreme differences in behavior^66,67^, microbiability offers a new opportunity to define novel contributory mechanisms through the gut-brain axis.

**Figure 6:**
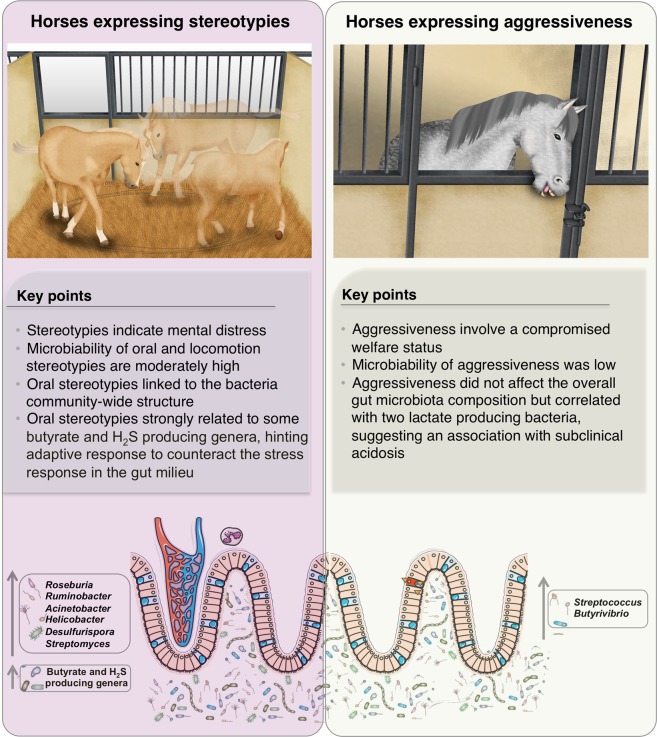
Behavior indicators relate to gut microbiota composition. We show that behaviors reflecting welfare deterioration such as stereotypies and aggressiveness are associated to the gut microbiota composition. On one hand, stereotypies, special oral stereotypies, were significantly linked to the overall gut microbiota structure and to single taxa. Oral stereotypies might presumably alter central nervous system, stress hormone release and feed intake behavior, modifying the gut milieu and the microbiota composition. Butyrate and H_2_S producing bacteria might confer to the host an adaptive response to reverse the potential negative effects of oral stereotypies. Reinforcing this idea, the microbiability of stereotypies was moderately high, suggesting that gut microbiota might be considered as an important metabolic organ with relevance to horse behavior. Written permission for publication of the drawing in the figure has been taken. On the other hand, aggressiveness, which reflects a compromised welfare status significantly correlated with two lactate producing bacteria, hinting that higher expression of aggressiveness lead to a subclinical acidosis.

Interestingly, our results showed that specific bacterial taxa (or their metabolites) were significantly linked to behaviors likely reflecting welfare deterioration. The strongest effect was observed in animals experiencing oral stereotypies. Levels of *Roseburia*, a butyrate-producing genus were among the core microbiota, positively correlated to the prevalence of oral stereotypies, along with amylolytic (*e.g. Ruminobacter)* and fibrolytic proteobacteria (*e.g. Acinetobacter*) or hydrogen sulfide (H_2_S)-producing genera (*e.g. Desulfurispora* and *Helicobacter*). Accumulating evidence shows that *Roseburia* exerts beneficial effects on host behavior through its anti-inflammatory activity within the gut^68^, whereas H_2_S, a gasotransmitter that protects the central nervous system^69^, has been shown to mitigate chronic restraint stress induced depression behavior in rats. A synergistic effect could exist between butyrate- and H_2_S producing bacteria, conferring to the host adaptive responses to reverse or counteract the potentially negative effects of oral stereotypies, including the bloom of potential pathobionts such as *Helicobacter* (which is known to be directly affected by norepinephrine secretion^64^), and modifications in the environmental characteristics of the intestinal milieu through the autonomic nervous system (*e.g*. gut motility, intestinal transit and secretion, gut permeability and hormone secretion; Fig. 6). Additionally, the positive correlation observed between locomotion stereotypies (which also reflects frustration at not being able to satisfy natural needs such as social contact, free movement and a continual intake of a fibrous diet^70^) and *Streptomyces* genus, a riboflavin transporter^71^, may very well reflect the functional adaptation for antioxidative defense in the gut. Additionally, two lactate producing bacteria correlated to aggressiveness, specifically, *Streptococcus* and *Butyrivibrio* genera. Impairment of the lactic acid metabolic pathway has been associated with the overgrowth of lactic acid tolerant bacteria and aggressiveness behavior in rats^72^ and acidosis in ruminants^73^, reflecting the potential bidirectional interactions within the brain-gut-microbiota axis (Fig. 6). Yet without clinical acidosis observed (fecal pH ranged within normal values); lactic acid bacteria associated with the levels of aggressiveness in horses should be validated in targeted experiments. The type of bedding material might also exacerbate aggressiveness. Seemingly, the interaction between gut microbiota and bedding material could be due to the higher fiber intake of animals housed in stalls on straw bedding as well as the community of microorganisms in the straw environment compared to those on non-straw bedding. In line with this, a recent study in pigs suggested that chopped straw bedding enriches the diet with non-soluble polysaccharides and seemingly positively selects for the members of Bacteroidetes and Fibrobacteres^74^. Importantly, straw bedding has been suggested to reduce aggressiveness in horses as it facilitates lying down, exploration, increased time during which the horse is occupied with feeding compared to those housed in boxes on shavings or wood pellets^36^. Beyond these variables outlined by Ruet *et al*.^36^, straw bedding may also reduce aggressiveness through gut microbiota, which communicate with the central nervous system through at least three interacting systems involving nervous, endocrine, and immune signaling mechanisms^75^.

Overall, this study provided an extended view of the sport horse gut microbiota and supported the hypothesis that bacterial taxonomic compositions were widely divergent between individuals: greater inter-individual than intra-individual variations were observed. This proportion was higher the rarer a microbial taxon was in the horse population.

Interestingly, building upon rich metadata, we identified currently unknown extrinsic and intrinsic variables affecting microbiota divergence, including equitation factors, straw bedding and host variables related to hematology and behavior impairment, with an overall effect size of 32%. In light with our results, the volume of physical exercise coupled with the expression of behaviors associated with welfare impairment (such as stereotypies, hypervigilance and aggressiveness) could reflect the underlying interaction between the gut microbiota and gut-brain axis. On one hand, physical or mental stress induced by intense exercise in horses could widely modified the eubiosis. On the other hand, gut microbiota could have influenced the central nervous system through nervous, endocrine and immune signaling mechanisms. Moreover, the microbiability, which is the contribution of the microbiome to the overall phenotype variance, for oral and locomotion stereotypies were 24.2% and 16.2%, respectively. We are still in the infancy of understanding the efficacy of modifications of the gut microbiota when utilizing antimicrobials, probiotics prebiotics, microbial transplantation or other interventions, but it is likely that those traits having higher microbiability could be easily improved or predicted using microbial data or modulating the microbiome.

Given the potential interaction between the gut microbiota and gut-brain axis upon stressful conditions, we caution the need to specifically evaluate how the gut microbiota or even their metabolites interact directly with stress hormones in peripheral tissues such as the mucosal layer of the gastrointestinal tract of equine athletes. Further knowledge regarding the role of gut microbiota in modulating excitatory and inhibitory neurotransmitters (*e.g*. serotonin, GABA and dopamine) and neurotransmitter-like substances in response to exercise-induced stress and its possible effects on the health and performance of the elite athletes is also required.

## Methods

### Animal cohort

The animal cohort and criteria of inclusion have been previously described by Massacci *et al*.^30^. Briefly, the present study included a cohort of 185 healthy sport horses (6 males, 50 mares and 129 geldings; age: 10.51 ± 3.47; Supplementary Table S1). Horses were recruited from a cohort of 376 individuals housed in the French National Ridding School, in Saumur (France). Out of 376 individuals, 189 were used for the study by Massacci *et al*.^30^, 185 individuals were used for the present one, while a total of 180 individuals were shared by both studies. As stated in our previous study^30^, for successful participation in the experiment, we required the following criteria: (i) written informed consent from owners; (ii) the horses were kept in the same box for at least six months prior of the beginning of the study (October 2016) and until the end of the study (June 2017); (iii) feces and blood samples collected at both time points, October 25th, 2016 (T1) and June 19th, 2017 (T2); (iv) absence of gastrointestinal disorders during the four months prior to sampling points; (v) absence of antibiotic treatments or immune modulating agents within four months prior to sampling points and absence of anthelmintic medication within 60 days before the sampling points, and (vi) data registration about concentrate composition and intake throughout the experiment, as well as equitation and behavior records.

### Individual variables

For each horse included in this study, we obtained the information related to age, sex, breed, body condition score and reproductive status. Body condition scores were determined for each animal using techniques developed by Caroll and coworkers^76^. Two trained technicians assigned a body condition score to every horse, and the averages of the two scores were calculated. The body condition scores ranged from 1 (very thin) to 5 (obese). The ideal average of body condition score ranges from 3 to 3.25. Individuals with a body condition score between 3.25 and 5 were classified as overweight.

### Equitation variables

We also recorded equitation factors such as the discipline (eventing, dressage, show-jumping or vaulting), and the hours being ridden, trained on the lunge or exercised with an automatic walker per week, as well as their levels of performance. Horses could have engaged in a low level of performance (amateur category), could be trained for high-level competitions (professional category) or have achieved the highest level of performance in international competitions or an equivalent level of training (expert category; Supplementary Table S1).

Additionally, we recorded the specialty. Briefly, Gala horses were high-level performers ascribed to high level of physical and mental engagement (with lots of transport and public shows). The Cadre Noir horses were those trained to become a regular high-level performer. They were submitted to regular and high level of physical and mental engagement, but without transport and public shows. Competition horses were medium-level performers and were subjected to punctual and high/medium level of physical and mental engagement (with transport and public competitions). Lastly, formation horses were those used for training candidate riders in the riding arena of the French National Riding School. They use to have low level of performance and they were permanently housed at the riding school, without expiring the stress of transport or public shows.

### Housing variables

Horses were restrictively housed in individual stalls, without access to paddocks or pastures. All stalls measured ± 9 m² and were cleaned six mornings out of seven mornings. We recorded information related to the type of bedding in the stall (*e.g*. straw, shavings or pellets). As outlined by Ruet *et al*.^36^, in the stalls, horses could have an open window allowing the observation of the external environment and other horses and/or a grilled window between two boxes allowing to benefit from visual and restricted tactile contact with the neighboring animal (Supplementary Table S1).

### Feeding variables

Horses received different quantities of concentrated rations, depending on their body condition score and physical activity, as previous specified in our study by Massacci *et al*.^30^. In addition, concentrate rations were distributed in three or four meals a day (Supplementary Table S1). In all cases, for each individual, the type and quantity of concentrate was kept constant throughout the experiment. Automatic drinkers provided water *ad libitum*, and all horses were fed with hay (9 kg in two meals per day), regardless of their needs. The detailed dietary records along the study were performed as detailed by Massacci *et al*.^30^. For each animal, the proportion and type of cereals and commercial feed supplements were recorded each week and carefully reviewed by the research staff. The cereals (mainly based on barley) and the commercial feed supplements were considered as concentrates. As specified elsewhere^30^, around 97% of the individuals received the same commercial concentrate (Flaked Royal Horse, Saint-Nolff, France) along the experiment consisting of flaked barley, alfalfa, flaked corn, soybean teguments, wheat straw, sugar beet pulp, molasses, extruded soybeans, rapeseed oil, flax oil, salt and a mineral and vitamin mix. The mineral and vitamin mix contained Ca (28.5%), P (1.6%), Na (5.6%), vitamin A (10,000 IU), vitamin D3 (1,500 IU), vitamin E (200 IU), vitamin B1 (12 mg/kg), cobalt carbonate (0.3 mg/kg), cupric sulfate (25 mg/kg), calcium iodate (0.4 mg/kg), iron sulfate (90 mg/kg), manganese sulfate (50 mg/kg), sodium selenite (0.30 mg/kg), and zinc sulfate (80 mg/kg).

The average daily intakes of macronutrients from cereals consumed were calculated based on the INRA food composition database for horses^77^, whilst the daily intakes of macronutrients listed on the commercial feed supplements were calculated according to the detailed descriptions of the respondents^13^. The net energy and digestible protein, expressed respectively in “Unités Fourragères Cheval” (UFC– horse feed units) and in “Matières Azotées Digestibles Cheval”(MADC) per Kg of dry matter, were also estimated based on INRA tables^78^. The UFC value of a feed is equal to the ratio between the net energy content of feed and the net energy content of barley^78^. The total macronutrients intake was then estimated by multiplying the frequency of the ingredient consumption by weight of an estimated average portion and nutrient content of the ingredient in question^13^ (Supplementary Table S1).

### Clinical variables

Parasite diagnosis was estimated through fecal egg counts (FEC, eggs per gram of wet feces). FEC was carried out using a modified McMaster technique^79^ on 5 g of feces diluted in 70 mL of NaCl solution with a density of 1.2 (sensitivity of 50 eggs/g).

None of the horses received antibiotic or anti-inflammatory treatment during the experiment, and diarrhea, clinical abnormalities (including colic) or fever was not detected in any horse.

### Assessment of the behavioral indicators using scan sampling during the experiment

The assessment of the behavioural indicators is detailed by Ruet *et al*.^36^. Briefly, four independent behavioral indicators reflecting compromised welfare state were recorded during the experiment: stereotypies, aggressive behaviors, and the occurrence of the “withdrawn posture” indicating unresponsiveness to the environment and the alert posture reflecting hypervigilance. Stereotypies included oral behaviors such as crib biting or locomotion behaviors such as weaving, nodding and head bobbing. Aggressiveness included a continuum of behaviors from ears pinned backward to biting, or kicking. The unresponsiveness to the environment was identified through a particular posture, called the “withdrawn posture”, where horses stood with the neck horizontal at the same level as back, with fixed stare, ears and head position. Finally, an internal state of hypervigilance was identified through the alert posture, described as elevated neck and ears pricked forward and looking intensely the environment. All behavior variables were retained as continuous variables, allowing the frequency of expression of these four indicators to be studied. Each horse was observed for five scans per day, on 50 non-consecutive days distributed over an eight-month period. Observations were equally distributed across times of the day. The average number of total scans analyzed per subject was 200 ± 18. The frequency of scans recorded for each behavioral indicator was calculated from the total number of observations per horse (Supplementary Table S1).

### Ethical statement

The local animal care and use committee reviewed and approved the study protocol (CEEA Val de Loire; reference: 2019012211274697.V4-18939, dated March 29th 2019). All protocols were conducted in accordance with EEC regulation (no 2010/63/UE) governing the care and use of laboratory animals, which has been effective in France since the 1st of January 2013. In all cases, owners and riders provided their informed consent prior to the start of study procedures with the animals.

### Weather data

Daily precipitation and temperatures were recorded at a meteorological station located 10 km from the French national riding school.

### Blood sampling and measurements

Blood samples were collected at T1 and T2 sample points. Whole blood samples were taken in EDTA and lithium heparin tubes (BD Vacutainer, 10 mL) for hematological assays. Blood was stirred for 15 min at room temperature to facilitate oxygenation. The erythron was assessed from peripheral blood samples by calculation the number of circulating red blood cells (RBC), hemoglobin concentration (HB), packed cell volume (PCV), volumetric indices, such as mean corpuscular volume (MCV), mean corpuscular hemoglobin (MCH) and mean corpuscular hemoglobin concentration (MCHC). The leukon was assessed from data derived from total and differential count of white blood cells (WBC) and the analysis of WBC morphology. Different WBCs were analyzed, including leucocytes (lymphocytes (LYM), monocytes (MON), neutrophils (NEU), basophils (BAS) and eosinophils (EOS)). The total blood cells were counted with a MS9-5 Hematology Counter (digital automatic hematology analyzer, Melet Schloesing Laboratories, France). Measurements values are reported in Supplementary Table S1.

### Fecal sampling

Fecal samples were collected from the rectum at T1 and T2 sample points, as described in our previous studies^13,30^. Fecal samples were collected because they provide a reasonable profile of the microbiota luminal composition of the healthy equine large intestine^16^.

Approximately 10 g of feces were collected from the center of several fecal balls, avoiding collection of fecal material that was touching the veterinarian globes. Fecal aliquots for microbiota analysis were immediately snap-frozen in liquid nitrogen and stored at -80 °C until DNA extraction, whereas fecal aliquots to measure the fecal pH were immediately sent to the laboratory.

### Fecal pH

The pH in the feces was determined after 10% fecal suspension (wt/vol) in saline solution (0.15 M NaCl solution; Supplementary Table S1).

### Microorganisms DNA extraction from fecal samples

Total DNA was extracted from aliquots of frozen fecal samples (200 mg), using E.Z.N.A. Stool DNA Kit (Omega Bio-Tek, Norcross, Georgia, USA). The DNA extraction protocol was carried out according to the manufacturer’s instructions (Omega Bio-Tek, Norcross, Georgia, USA). DNA was then quantified using the Qubit dsDNA HS assay kit (Thermo Fisher Scientific, Waltham, MA, USA).

### V3–V4 16 S rRNA gene amplification

The V3-V4 hyper-variable regions of the 16 S rRNA gene were amplified with two rounds of PCR as previously described in our lab^6,13,14^. The concentration of the purified amplicons was measured using Nanodrop 8000 spectrophotometer (Thermo Fisher Scientific, Waltham, MA, USA) and the quality of a set of amplicons was checked using DNA 7500 chips onto a Bioanalyzer 2100 (Agilent Technologies, Santa Clara, CA, USA). All libraries were pooled at equimolar concentration in order to generate equivalent number of raw reads with each library. The final pool had a diluted concentration of 5 nM to 20 nM and was used for sequencing. Amplicon libraries were mixed with 15% PhiX control according to the Illumina’s protocol. For this study, one-sequencing run was performed using MiSeq reagent kit v2 (2 ×250 output; Illumina, San Diego, CA, USA).

### V3–V4 16 S rRNA gene sequencing and data pre-processing

Sequences were processed using the version 1.9.0 of the Quantitative Insights Into Microbial Ecology (QIIME) pipeline^80,81^ and by choosing the open-reference OTU calling approach^81^, as described elsewhere^13,30^.

First, forward and reverse paired-end sequence reads were collapsed into a single continuous sequence according to the ‘fastq-join’ option of the ‘join_paired_ends.py’ command in QIIME. The fastq-join function allowed a maximum difference within overlap region of 8%, a minimum overlap setting of 6 bp and a maximum overlap setting of 60 bp. The reads that did not overlap (~20% of the total) were removed from the analysis. Anomalously joined reads, reads that were too short or too long, were excluded according to the expected size of each targeting region (430–469 bp for region V3–V4). The retained sequences were then quality filtered. De-multiplexing, primer removal and quality filtering processes were performed using the ‘split_libraries’_fastq.py command in QIIME. We applied a default base call Phred threshold of 20, allowing maximum three low-quality base calls before truncating a read, including only reads with >75% consecutive high-quality base calls, and excluding reads with ambiguous (N) base calls.

Subsequently, the sequences were clustered into OTUs against the GreenGenes database (release 2013-08: gg_13_8_otus)^82^ by using the uclust^83^ method at a 97% similarity cutoff. The filtering of chimeric OTUs was performed by using Usearch version 6.1^84^ against the GreenGenes reference alignment^82^. A phylogenic tree was generated from the filtered alignment using FastTree^85^. Because the relatively short read length of Illumina-generated sequences could reduce the resolution of taxonomic annotation against the GreenGenes reference database, the resulting OTU representative sequences were then searched against the Ribosomal Database Project naïve Bayesian classifier (RDP 10 database, version 6) database, using the online program SEQMATCH (http://rdp.cme.msu.edu/seqmatch/seqmatch_intro.jsp). Singletons were discarded from the dataset using the ‘filter_otus_from_otu_table.py’ script in QIIME. The Phyloseq^86^, Vegan^87^ and microbiome R packages were used for the detailed downstream analysis. The minimum sampling depth in our data set was 10,000 reads per sample. The OTU tables were rarefied to the sample containing the lowest number of sequences (n = 10,000). The minimal sequencing depth of 10,000 was sufficient for accurately profiling bacterial composition, as predicted by the calculation of the rarefaction curve for observed richness and Shannon index (which accounts for both abundance and evenness).

OTU counts per sample and OTU taxonomical assignments are available in Supplementary Table S2. Data was aggregated at genus, family, order, class and phyla levels throughout the taxonomic-agglomeration method in Phyloseq R package, which merges taxa of the same taxonomic category for a user-specific taxonomic level. Relative abundance normalization was applied, which divides raw counts from a particular sample by the total number of reads in each sample.

### Real-time quantitative PCR (qPCR) analysis of bacterial, anaerobic fungal and protozoan loads

Concentrations of protozoa, anaerobic fungi and bacteria in fecal samples were quantified using a QuantStudio 12 K Flex real-time instrument (Thermo Fisher Scientific, Waltham, USA). Primers for real-time amplification of protozoa (FOR: 5′-GCTTTCGWTGGTAGTGTATT-3′; REV: 5′-CTTGCCCTCYAATCGTWCT-3′),

anaerobic fungi (FOR: 5′-TCCTACCCTTTGTGAATTTG-3′; REV: 5′-CTGCGTTCTTCATCGTTGCG-3′) and bacteria (5′-CAGCMGCCGCGGTAANWC-3′; REV: 5′-CCGTCAATTCMTTTRAGTTT-3′). Details on thermal cycles and the creation of standard curve for absolute quantification are reported in our previous study^13^. To generate quantification curves, purified DNA was quantified using the Qubit dsDNA HS assay kit (Thermo Fisher Scientific; Waltham, MA USA). This DNA was subsequently diluted serially by copy number and amplified using the 16 S rRNA, the 18 S rRNA or ITS1 qRT-PCR assays. The standard curve was included in each run. After each run, melting curve analysis was performed to the presence of the desired amplicon and to confirm the lack of primer dimers. In all cases, the melting curves analysis did not reveal any contamination due to genomic DNA or to non-specific amplification. Gel electrophoresis analysis of the PCR products also showed a single band of the expected size. The qPCR efficiencies covering the three amplicons were calculated in each run, as described by Plancade *et al*.^13^.

### Statistical analysis

#### Temporal dynamics of host and gut environment variables: blood hematological variables, fecal pH and fecal loads of fungi, protozoa and bacteria

The influence of time on blood hematological variables, fecal pH and fecal loads of anaerobic fungi, bacterial and protozoa was examined using generalized linear mixed models (GLMMs). GLMMs were run using ‘*lmer’* function for data normally distributed or data transformable to normality (*e.g*. log transformation) or using ‘*glmer’* function for data not normally distributed in lme4 R package. Time was included in the model as fixed effect, and horse was included in the model as random effect. Models were simplified using a backward deletion process based on Akaike’s information criterion (AIC), starting with a maximal model that included all predictors and their possible interactions. Additionally, differences between T1 and T2 were tested using the nonparametric Wilcoxon signed-rank test followed by Benjamini and Hochberg multiple test correction. An adjusted *p*-value <0.05 was considered as significant.

#### Description of the core microbiota

Similarly to Massacci *et al*.^30^, to characterize a set of microbes consistently present at T1 and T2, we used a detection threshold of 0.1% and a prevalence threshold of 99.9% (*e.g*. a given genera must be present in 99.9% of individuals in each time point with a relative abundance of at least 0.1%) using the microbiome^88^ R package.

#### Alpha diversity indices of the fecal microbiota and its temporal dynamics

The α-diversity indices were calculated using the Phyloseq and microbiome R packages from OTU and relative genera abundance tables, as described in our previous studies^13,30^. Briefly, the microbiome R package allowed us to study global indicators of the gut ecosystem state, including measures of evenness, dominance, divergences and abundance. All samples were normalized using the rarefy_even_depth function in the Phyloseq R package, which is implemented as an *ad hoc* means to normalize microbiome counts that have resulted from libraries of widely differing size.

The microbial α-diversity metrics between the two time sampling points were compared using normal linear mixed models (lme4 R package^89^) with time as fixed effect and subject as random effect. A normal (Gaussian) linear mixed model was fitted and the model fits were checked using residual plots. The scatter of residuals showed no pattern, suggesting that the data fit the model and hence conclusions drawn from the models were valid. Complementary, differences between T1 and T2 were tested using the nonparametric Wilcoxon signed-rank test followed by Benjamini and Hochberg multiple test correction. An adjusted *p*-value <0.05 was considered as significant.

#### Beta diversity of fecal microbiota and its inter-individual and temporal dynamics

To estimate β-diversity, un-weighted and weighted UniFrac distances, as well as Bray-Curtis dissimilarity, were calculated from the OTU and the genera relative abundance tables. The β-diversity was visualized using the NMDS through the Phyloseq and Vegan R packages.

The inter-individual variations in the gut microbiota composition was studied based on the conceptual framework of enterotypes, or more generically, community types^90^. According to this framework, the samples were clustered into bins based on their taxonomic similarity^91^. Briefly, clustering was performed with PAM^92^ using weighted UniFrac distance of the normalized OTUs counts at each time point. The optimal number of clusters or communities was chosen by the maximum average silhouette width, known as the silhouette coefficient (SC)^93^. The quality of those clusters or community types was assessed by the same measure, following the accepted interpretation that SC values above 0.5 indicate a reasonable clustering structure^94^.

The among-time differences in the gut microbiota composition were tested through complementary approaches: (i) the PERMANOVA; (ii) the ANOSIM and (iii) the SIMPER based on Bray-Curtis dissimilarity at the OTU and genera levels.

For the PERMANOVA, we tested the effects of time corrected by breed and sex on the variation of total dissimilarity between microbiota. Although breed exerts limited effects on the equine fecal microbiota^30^, it is recommended to account for breed variation as a potential confounding factor in studies linking microbiota differences to host phenotypes in horse. The significance of the effect of time was assessed in an *F*-test based on the sequential sum of squares estimated from a 10,000 permutations procedure. The significance threshold was chosen at adjusted *p-*value <0.05.

The ANOSIM was performed to compare within- and between-time similarity through a distance measure, to test the null hypothesis that the average rank similarity between samples within a time point was the same as the average rank similarity between samples belonging to different time points.

Lastly, the homogeneity of dispersions of microbiota composition between time points was tested through the Whittaker’s index using the multivariate analyses of the homogeneity of group dispersion (the ‘*betadisper*’ function of the Vegan R package), that is, the distance of individual time points from the centroid of their time point. Moreover, to assess whether the microbial homogeneity across time was statistically different, we performed an ANOVA using the ‘*aov*’ function in R followed by the *post hoc* Tukey-Kramer at 0.95 and the multiple test correction of Benjamini and Hochberg (adjusted *p*–value <0.05).

#### The contribution of each genus to the ecosystem temporal dynamics

Differentially expression analysis based on the negative binomial distribution using Wald test was applied through DESeq2^95^ R package to test for differential genera abundances between the two time points. DESeq2 assumes that counts can be modeled as a negative binomial distribution with a mean parameter, allowing for size factors and a dispersion parameter. Next to the time factor, the horse‐dependency were included in the generalized linear model. The *p*-values were adjusted for multiple testing using the Benjamini and Hochberg procedure.

SIMPER analysis was performed to show which taxa contributed to this difference the most.

Lastly, the PLS-DA was used to identify the key genera responsible for the differences in the gut community between the two time points using the mixOmics^96^ R package.

#### Temporal associations between gut microbiota, host and environmental variables

To eliminate the inter-individual variability between the two time points, the Δ values of gut microbiota composition (based on the relative genera abundance table), host variables (*e.g*. hematological profile and behavior indicators) and gut phenotypes (*e.g*. fecal pH, fecal microorganism loads) were computed. In all cases, deltas were calculated as individual differences between T2 (end of the study) and T1 (baseline values).

Next to the time varying variables, we also considered host and environmental variables whose values did not change between the two time points, *e.g*. sex and breed. The environmental variables with time-invariant values considered in the model were the presence or absence of a grilled window between boxes, the bedding material, the mean quantity of concentrated feed offered and the number of meal per day, the discipline and specialty along with the level of performance, the number of competing events performed during the study, the average time spent being ridden and being trained on the lunge/using an automatic walker each week.

We tested for associations between the genus-level community composition and host and environmental variables using the NMDS based on Bray-Curtis dissimilarity ordination method followed by the *envfit* function^97^ in the vegan R package, with 10,000 permutations and Benjamini and Hochberg multiple testing correction. An adjusted *p*-value <0.05 was considered as significant. This function performs a multivariate analysis of variance (MANOVA) and linear correlations for categorical and continuous variables, respectively, and enables the selection of combined covariates with strongest correlation to microbiota variation.

Cross correlation between genera and host phenotypes was performed using the ‘*associate*’ function in the microbiome R package. The adjusted *p*-values are provided by the default method in the ‘*cor.test*’ function. Complementary, sparse CCA, a method well-suited to both exploratory comparisons between samples and the identification of features with interesting variation, was implementation from the PMA R package^98^. Lastly, for differential abundance comparisons between host variables, we used DESeq2 R package. All comparisons were performed on genus level. DESeq2 comparisons were run with the parameters fitType = “parametric” and sfType = “poscounts”.

### Behavior phenotypes and microbiability

The microbiability (m^2^), which is the contribution of the microbiome to the overall phenotype variance^99^, was calculated as follows:

$${m}^{2}={\sigma }_{m}^{2}/({\sigma }_{H}^{2}+{\sigma }_{e}^{2})$$

where ${\sigma }_{m}^{2}$ is the microbiome variance, ${\sigma }_{H}^{2}$ is the genetic variance of the host and ${\sigma }_{e}^{2}$ represents the residual variance. The term m^2^, and the way it is estimated, is analogous to heritability as it depends on the partitioning of observed variation into components that reflect unobserved host genetic and environmental variables. Given its definition as a ratio of variance components, the value of microbiability always lies between 0 and 1. If m^2^ = 1, all variation of the phenotype in the population is due to differences or variation between microbiomes (*e.g*., there is no environmental variation). The higher the microbiability, the greater is the microbiome control on the phenotype.

The proportion of behavior and blood variables phenotypic variance explained by the bacterial community was estimated through a Bayesian mixed model implemented using the BGLR R package^100^:

$${y}_{j}=1{\mu }+bree{d}_{j}+disciplin{e}_{j}+tim{e}_{j}+b+{e}_{j}$$

where *y* is the phenotype vector (*e.g*. hypervigilance, unresponsiveness, aggressiveness), 1*μ* is the intercept, and *breed*, *discipline* and *time* represent the fixed effects. Lastly, *b* represents the microbial relationship matrix based on the variance-covariance matrix from the Cumulative Sum Scaling^101^ normalized and log transformed OTU table^102^. A prior uniform distribution was assumed for the fixed effects (breed, discipline and time). The *ε* is the residual term. The model was run using a Gibbs sampler with 30,000 iterations and a burn-in of 2 000 rounds as described by Ramayo-Caldas *et al.*^103^.

### Horse drawings

Horse images were created with Adobe Illustrator CS6 (v.16).

## Supplementary information

Supplementary Information.

Supplementary Information2.

Supplementary Information3.

Supplementary Information4.

Supplementary Information5.

Supplementary Information6.

Supplementary Information7.

Supplementary Information8.

Supplementary Information9.

Supplementary Information10.

## Data Availability

This Targeted Locus Study project has been deposited at DDBJ/EMBL/GenBank under the accession KDDC00000000. The version described in this paper is the first version, KDDC01000000. The bioproject described in this paper belongs to the BioProject PRJNA543287. The corresponding BioSamples accession numbers were SAMN11666402 to SAMN11666784.
