# Supplementary figures and images for: Priming for welfare: gut microbiota is associated with equitation conditions and behavior in horse athletes
Source: Sci Rep. 2020 May 20;10:8311. doi: 10.1038/s41598-020-65444-9 (PMC7239938; doi:10.1038/s41598-020-65444-9)

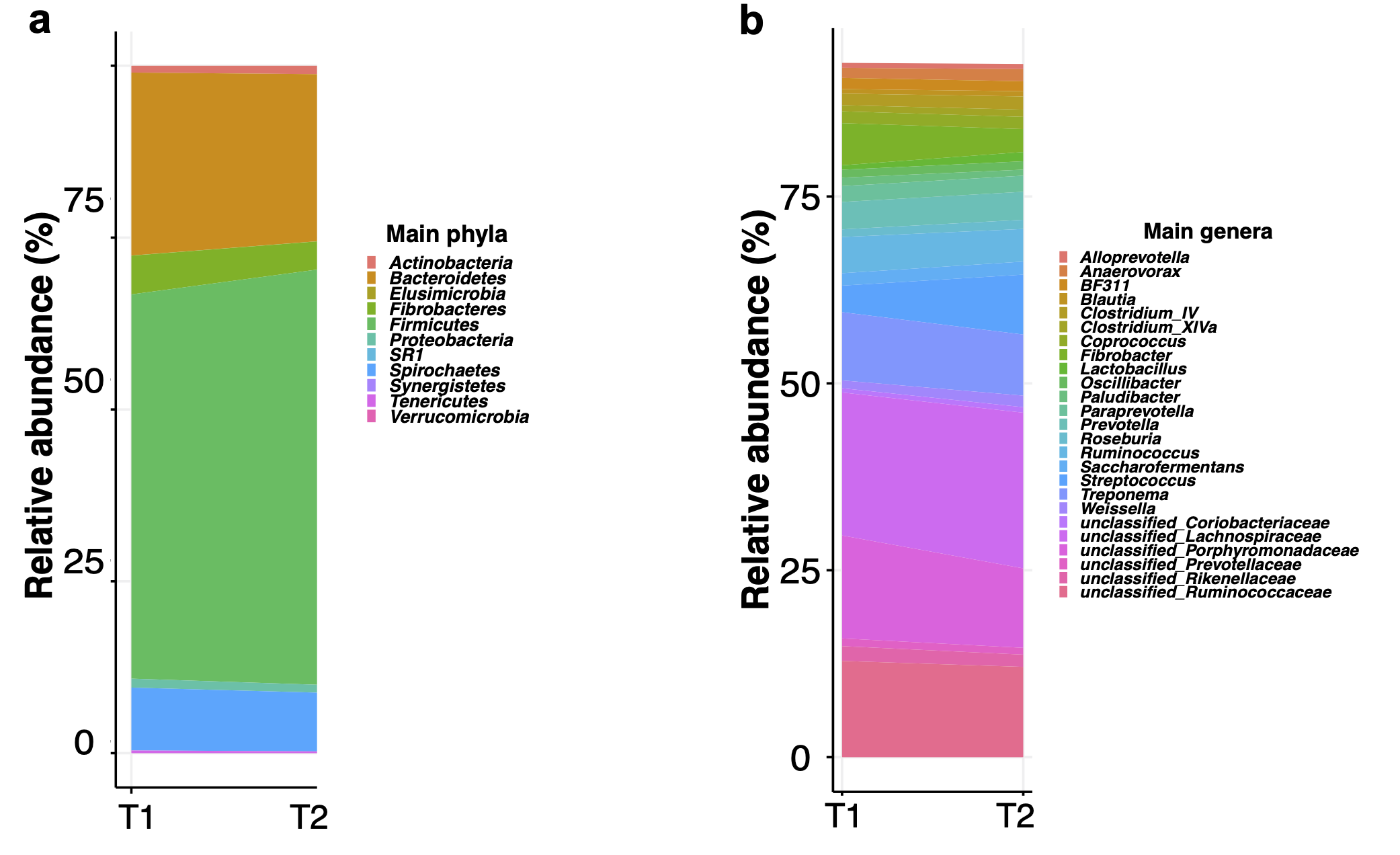

Supplement: Supplementary file 2 — Supplementary Information2. [file 41598_2020_65444_MOESM2_ESM.tiff]

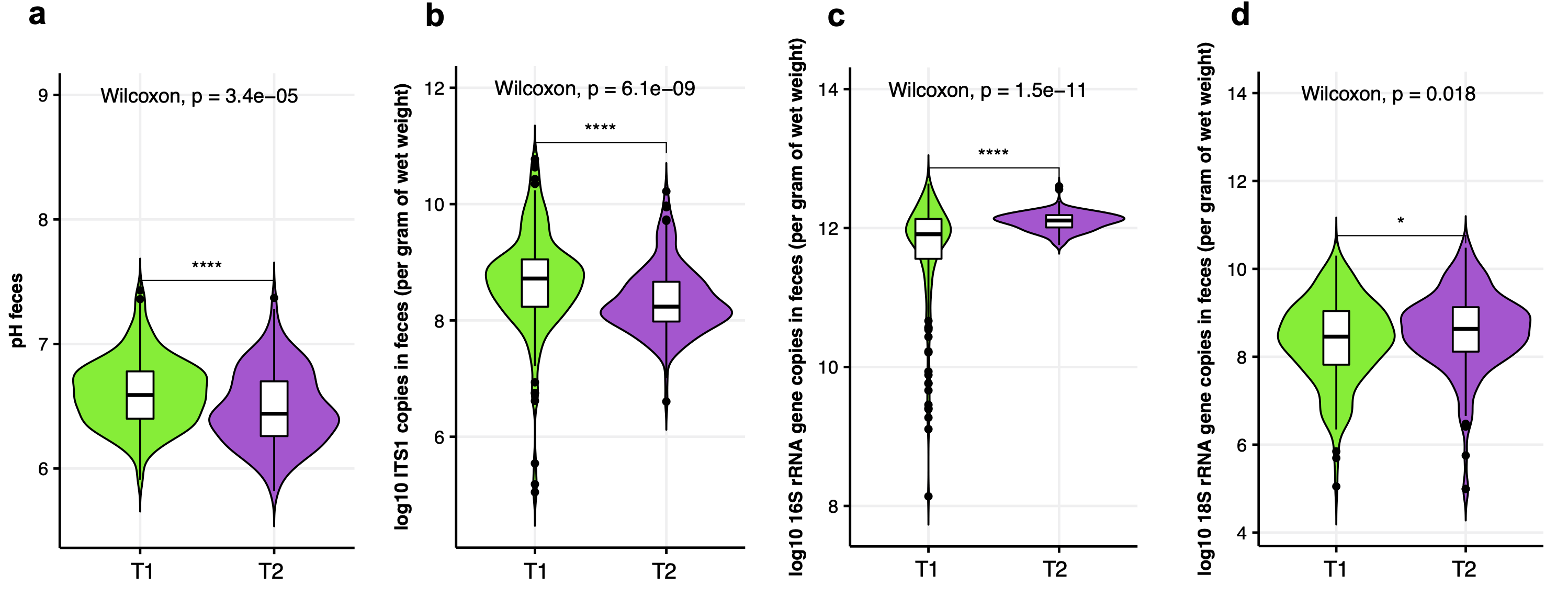

Supplement: Supplementary file 3 — Supplementary Information3. [file 41598_2020_65444_MOESM3_ESM.tiff]

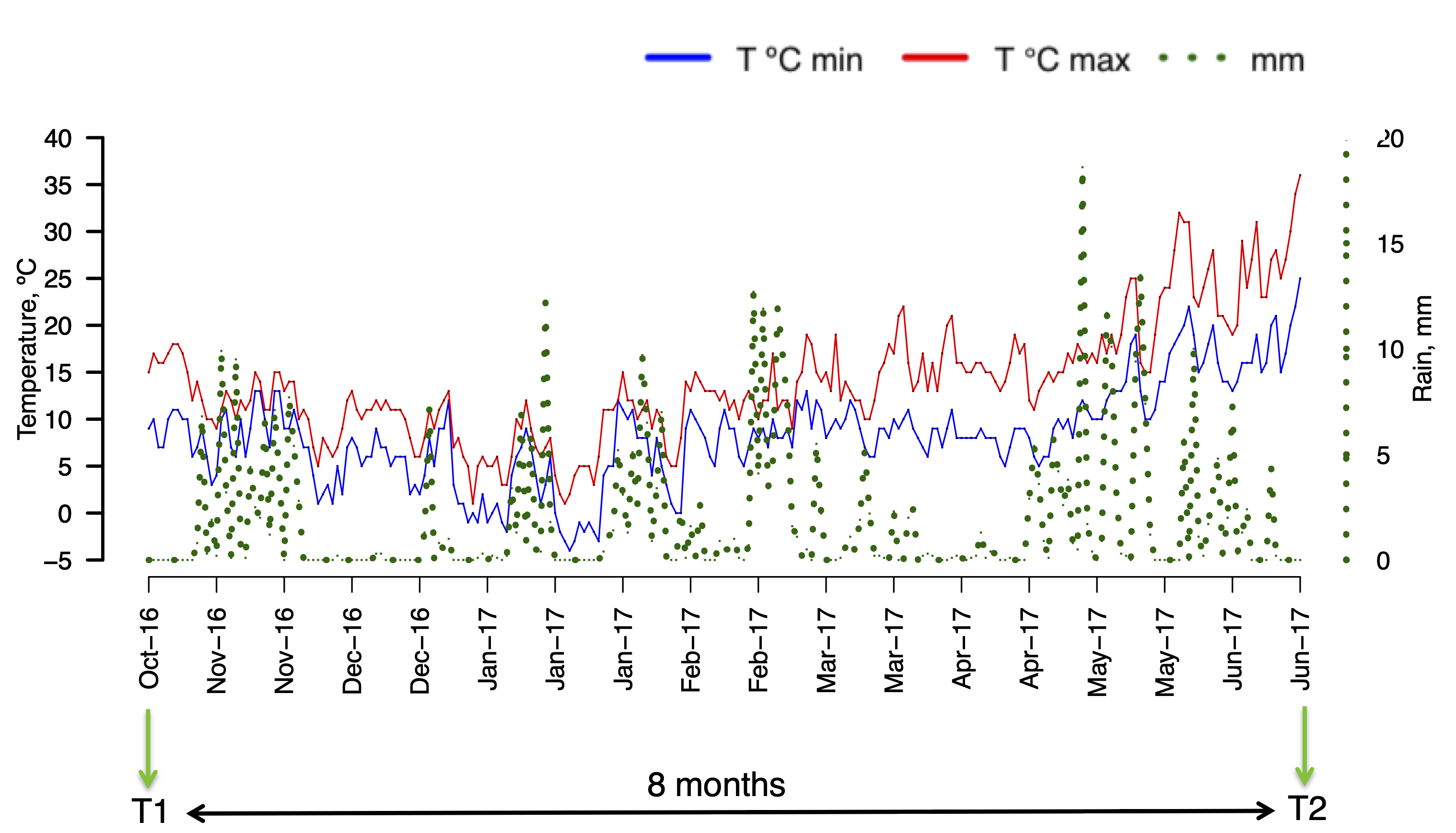

Supplement: Supplementary file 4 — Supplementary Information4. [file 41598_2020_65444_MOESM4_ESM.tiff]

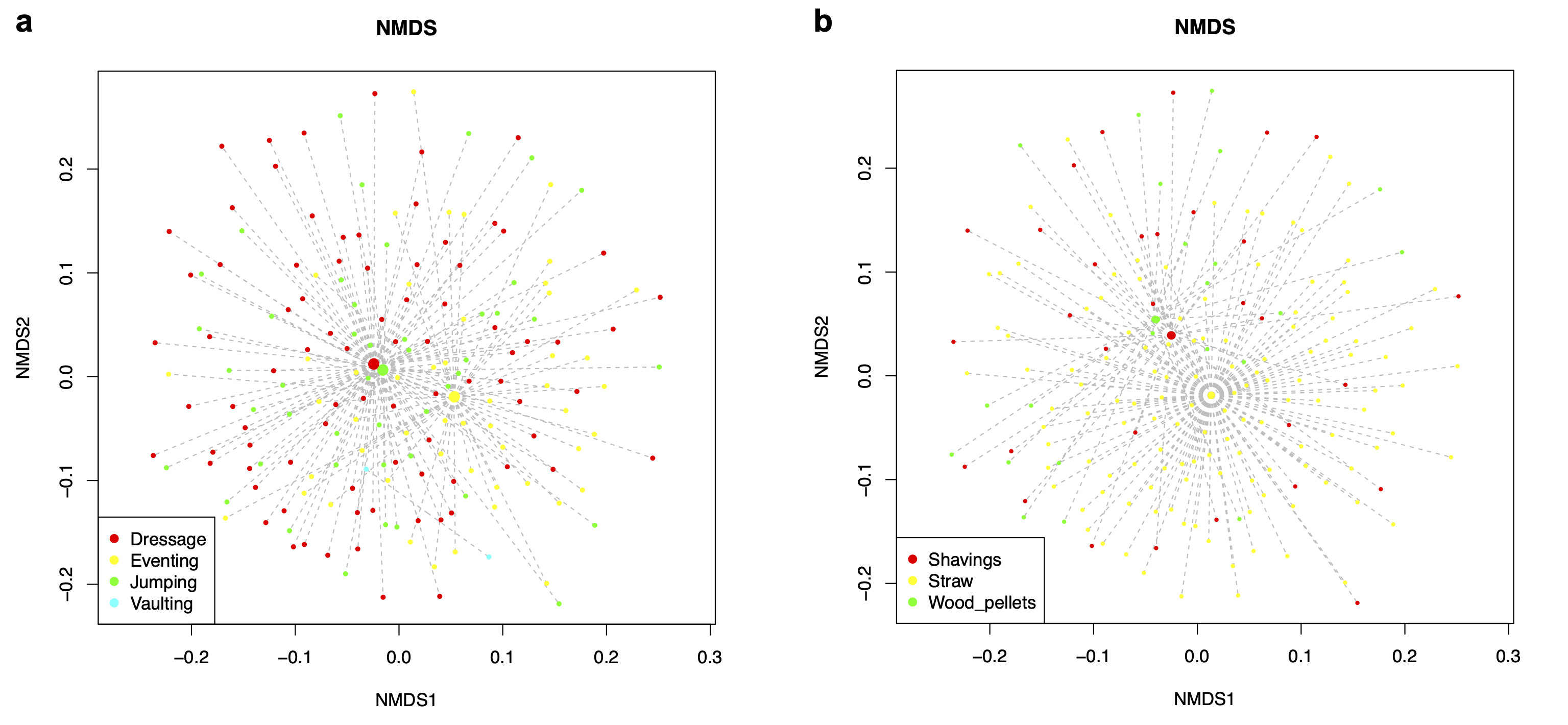

Supplement: Supplementary file 5 — Supplementary Information5. [file 41598_2020_65444_MOESM5_ESM.tiff]
